# Validation of the Toronto recurrence inference using machine-learning for post-transplant hepatocellular carcinoma model

**DOI:** 10.1038/s43856-025-00994-5

**Published:** 2025-07-09

**Authors:** Zhihao Li, Itsuko Chih-Yi Chen, Leonardo Centonze, Christian T. J. Magyar, Woo Jin Choi, Tommy Ivanics, Grainne M. O’Kane, Arndt Vogel, Lauren Erdman, Luciano De Carlis, Jan Lerut, Quirino Lai, Vatche G. Agopian, Neil Mehta, Chao-Long Chen, Gonzalo Sapisochin

**Affiliations:** 1 HBP & Multi-Organ Transplant Program, University Health Network, Toronto, Canada; 2 Department of Surgery, Kaohsiung Chang Gung Memorial Hospital, Kaohsiung, Taiwan; 3 Department of General Surgery and Transplantation, Niguarda Ca’ Granda Hospital, Milan, Italy; 4 Clinical and Experimental Medicine PhD Program, University of Modena and Reggio Emilia, Modena, Italy; 5 Department of Visceral Surgery and Medicine, Inselspital, Bern University Hospital, University of Bern, Bern, Switzerland; 6 Department of Medical Oncology, St. Vincent’s University Hospital, Dublin, Republic of Ireland; 7 Wallace McCain Centre for Pancreatic Cancer, Princess Margaret Hospital, Toronto, Canada; 8 Division of Gastroenterology and Hepatology, University Health Network, Toronto, Canada; 9 Department of Gastroenterology, Hepatology, Infectious Diseases and Endocrinology, Hannover Medical School, Hannover, Germany; 10 The Center for Computational Medicine, The Hospital for Sick Children, Toronto, ON Canada; 11 James M. Anderson Center for Health Systems Excellence, Cincinnati Children’s Hospital Medical Center and University of Cincinnati School of Medicine, Cincinnati, OH USA; 12 School of Medicine and Surgery, University of Milan-Bicocca, Milan, Italy; 13 Institut de Recherche Expérimentale et Clinique, Université catholique de Louvain, 10 Avenue Hippocrate, 1200 Brussels, Belgium; 14 General Surgery and Organ Transplantation Unit, Sapienza University of Rome, Rome, Italy; 15 Division of Liver and Pancreas Transplantation, Department of Surgery, Dumont-UCLA Transplant and Liver Cancer Centers, David Geffen School of Medicine at University of California, Los Angeles, CA USA; 16 Division of Gastroenterology, Department of Medicine, University of California San Francisco, San Francisco, USA

**Keywords:** Surgical oncology, Hepatocellular carcinoma

## Abstract

**Background:**

Organ shortages require prioritizing hepatocellular carcinoma (HCC) patients with the highest survival benefit for allografts. While traditional models like AFP, MORAL, and HALT-HCC are commonly used for recurrence risk prediction, the TRIUMPH model, which uses machine learning, has shown superior performance. This study aims to externally validate the model.

**Methods:**

The cohort included 2844 HCC patients who underwent liver transplantation at six international centers from 2000-2022. The TRIUMPH model utilized a regularized Cox proportional hazards approach with a penalty term for coefficient adjustment. Discrimination was assessed using the c-index, and clinical utility was evaluated via decision curve analysis.

**Results:**

The most common liver diseases are hepatitis C (49%) and hepatitis B (27%). At listing, 84% meets the Milan criteria, and 91% are within criteria at transplant. Median model for end-stage liver disease score is 10 (IQR:8–14), alpha-fetoprotein level 8 ng/mL (IQR:4–25), and tumor size 2 cm (IQR:1.1–3.0). Living donor grafts are used in 24% of cases. Recurrence rate is 9.1% with a median time to recurrence of 17.5 months. Recurrence-free survival rates at 1/3/5 years are 95.7%/89.5%/87.7%, respectively. The TRIUMPH model achieves the highest c-index (0.71), outperforming MORAL (0.61, *p* = 0.049) and AFP (0.61, *p* = 0.04), though not significantly better than HALT-HCC (0.67, *p* = 0.28). TRIUMPH shows superior clinical utility up to a threshold of 0.6.

**Conclusions:**

The TRIUMPH model demonstrates good accuracy and clinical utility in predicting post-transplant HCC recurrence. Its integration into organ allocation could improve transplantation outcomes.

## Plain Language Summary

Liver transplants are a limited resource, so doctors must prioritize patients with liver cancer (HCC) who are most likely to benefit. Some traditional tools exist to help predict cancer recurrence, but a new tool called TRIUMPH, which uses machine learning, may work better. This study looked at 2844 HCC patients who received liver transplants at six international centers. Most had hepatitis-related liver disease, and 9% experienced cancer recurrence after transplant. The TRIUMPH model more accurately predicted which patients would stay cancer-free, outperforming most traditional tools. It also proved more helpful in guiding treatment decisions. These findings suggest TRIUMPH could help doctors make better use of available livers by selecting patients with the best long-term outcomes. This could lead to improved survival and more efficient organ allocation worldwide.

## Abstract (web summary)

Li et al. aims to externally validate the TRIUMPH model, a machine learning model previously established to predict post-transplant HCC recurrence. It demonstrates good accuracy and clinical utility, superior to pre-existing models including MORAL, AFP, and HALT-HCC.

## Introduction

Liver transplantation (LT) represents the optimal treatment for patients with early-stage hepatocellular carcinoma (HCC)^1,2^. However, due to organ shortages, allocating allografts for HCC patients requires careful consideration of patient-specific survival benefits, alternative treatment options, and the equitable distribution of donor organs.

The current selection criteria aim to exclude futile LT in patients with tumor characteristics linked to a high risk of post-transplant recurrence^3^. Although the Milan criteria^4^ have long been the standard for selecting HCC patients for LT, more sophisticated risk prediction models have emerged over the years. These models, including AFP^5^, MORAL^6^, and HALT-HCC^7^, incorporate not only morphological markers but also some biomarkers such as serum alpha-fetoprotein (AFP) and the neutrophil-to-lymphocyte ratio. To improve risk prediction further, incorporating radiologic and AFP responses to bridging or downstaging treatments, along with new biomarkers, represent an unmet need^8–10^. As HCC management evolves and clinical data expands, incorporating additional relevant risk factors could enhance prediction accuracy. However, a key challenge in developing such a calculator is the limitation of standard statistical methods in handling numerous variables and their complex interactions.

Machine learning, as a leading technology of the century, offers a powerful tool for identifying complex patterns in large datasets with multiple variables—far beyond human capacity to process^11^. In the context of LT for HCC, machine learning could be leveraged to develop a highly accurate predictive calculator, enhancing decision-making for organ allocation^12^. This approach was utilized previously in developing the Toronto calculator^13^, hereafter called the TRIUMPH model.

This study externally validates the TRIUMPH model using a large international HCC liver transplant database, comparing its performance to conventional models (AFP, MORAL, HALT-HCC). We hypothesize that the machine learning-based model will show superior discrimination for post-transplant recurrence potentially enhancing organ allocation.

## Methods

### Study design and population

The external validation cohort consists of data from six prospectively maintained databases: two North American, three European and one Asian. Inclusion criteria were adult patients who received LT from January 2000 to November 2022 for a known HCC. Excluded were patients with intrahepatic cholangiocarcinoma or combined hepatocelluar-cholangiocarcinoma. This study has received institutional ethical approval from the University Health Network Research Ethics Board (REB#: 22-5019).

### Transplant listing eligibility and peritransplant management

Eligibility criteria and prioritization for organ allocation varied among different centers. In the United States, the UNOS system awards MELD exception points based on the Milan criteria. The national health institute in Taiwan adhered to the Milan criteria until 2006 and subsequently adopted the University of California, San Francisco (UCSF) criteria^14^. In Italy and Belgium, the criteria for transplant eligibility mandated adherence to the Milan criteria^15^ until 2008, after which the up-to-7 criteria^16^ were adopted.

Patients with an expected waitlist time of over 6 months underwent bridging therapy, which included transarterial chemoembolization, transarterial radioembolization, ablation, percutaneous ethanol injection, or radiotherapy. Patients who did not meet the eligibility criteria in their respective countries received locoregional therapy for downstaging. Some patients had undergone a previous liver resection intended as definitive treatment. In these patients, LT was performed either before recurrence due to high-risk factors (e.g. microvascular invasion) in the resection pathology, or as salvage therapy for recurrence after resection^17^.

Ultrasound and/or thoracoabdominal computed tomography was performed every 6 months for the first 3 years of follow-up. Analysis of alpha-fetoprotein (AFP) values was also performed every 6 months. Thereafter, computed tomography was performed annually or if symptoms occurred. UHN and UCSF have adjusted the extent and intensity of surveillance according to the RETREAT score^14^ since 2015. Diagnosis of tumor recurrence was based on imaging. A biopsy was performed if the image was inconclusive.

### Data collection and definition

Baseline clinical and demographic characteristics were recorded for each patient. Follow-up continued until either the patient’s death from any cause or the last follow-up date. The recurrence-free survival was determined from the date of transplantation to the date when the initial imaging confirmed tumor recurrence or patient death.

### Model comparison

Details on the development of the TRIUMPH model based on CoxNet analysis are provided in the previous paper^13^. Briefly, it is a regularized Cox proportional hazards model with a penalty term to regularize coefficients during fitting. Model discrimination was assessed using the c-index and compared against the AFP, MORAL, and HALT-HCC models, which were chosen on the basis of the previous publication^13^, for their use of pre-LT variables and focus on recurrence risk (AFP, MORAL) and post-transplantation survival (HALT-HCC). Model parameters were derived from the original publications (Table 1).

**Table 1:** Reported coefficients of the four models (TRIUMPH, HALT-HCC, MORAL, AFP)

TRIUMPH	Reported Coefficient
Age, per 1-year increase	−0.004
Etiology: other (non-viral, non-alcoholic, non-MAFLD)	−0.153
Number of bridging therapies, per 1-unit increase	0.228
log-AFP (before transplantation), per 1-unit increase (ng/mL)	0.191
Neutrophil count (before transplantation), per 1-unit increase (×10^9^/L)	0.025
Sodium (before transplantation), per 1-unit increase (mmol/L)	−0.010
Total tumor diameter (at listing), per 1-cm increase	0.041
Largest lesion size (at listing), per 1-cm increase	0.020
Largest lesion size (before transplantation), per 1-cm increase	0.020
Tumor burden score (before transplantation), per 1-unit increase	0.038
Within Milan criteria (before transplantation)	−0.060
HALT-HCC	
log-AFP (before transplantation), per 1-unit increase (ng/mL)	0.547
MELD-Na score (before transplantation), per 1-unit increase	0.077
Tumor burden score (before transplantation), per 1-unit increase	0.376
MORAL	
Maximum AFP ≥ 200 ng/mL (before transplantation)	0.318
Neutrophil-to-lymphocyte ratio ≥5 (before transplantation)	0.417
Maximum lesion size ≥3 cm (before transplantation)	0.265
AFP	
3 cm <maximum lesion size ≤6 cm (at listing)	0.069
Maximum lesion size >6 cm (at listing)	0.343
Lesion count ≥4 (at listing)	0.177
100 < AFP ≤ 1000 ng/mL (at listing)	0.170
AFP > 1000 ng/mL (at listing)	0.241

*AFP* Alpha-fetoprotein, *MAFLD* Metabolic Dysfunction-Associated Fatty Liver Disease, *MELD-NA* Model for End-Stage Liver Disease incorporating Sodium.

### Net benefit analysis

To evaluate and compare the clinical utility of the prediction models, we conducted a net benefit analysis using decision curve analysis. This method assesses a model’s clinical value by weighing the benefits of true positives against the harms of false positives, considering the threshold probability at which clinical decisions (e.g., treatment) are made. Net benefit was calculated across a range of plausible threshold probabilities using the following formula (Eq. 1):

1 $${Net\; Benefit}=\frac{{True\; Positives}}{n}-\frac{{False\; Positives}}{n}\times \frac{{Threshold\; Probability}}{1-{Threshold\; Probability}}$$

The model’s net benefit was plotted against a range of threshold probabilities and compared to the default strategies of intervening on all patients (‘treat all’) and intervening on no patients (‘treat none’, net benefit = 0). The model demonstrates clinical utility across the range of thresholds where its net benefit is superior to both default strategies, indicating its potential value for guiding clinical decisions.

### Statistical analysis

Categorical variables were reported as numbers and percentages, continuous variables as medians and interquartile ranges (IQR). Categorical variables were compared using the Chi-square test and continuous variables using the Mann-Whitney U test. Patient survival was estimated using the Kaplan-Meier method and groups compared with the log-rank test. One-sided z-tests were used to compare the TRIUMPH model with 3 competing models in order to test the alternative hypothesis that the TRIUMPH performs better than previously published algorithms. All statistical analysis was performed using R Studio software (R Foundation for Statistical Computing v4.4.1, 2024-06-14). We used the *survminer* package for survival analysis and *rmda* to assess the models’ net benefit in guiding treatment decisions.

### Reporting summary

Further information on research design is available in the Nature Portfolio Reporting Summary linked to this article.

## Results

### Characteristics of the validation cohort

A total of 2844 patients with HCC, confirmed by explant pathology, were included in the validation cohort. The median age was 59 years (IQR: 54-64), and 80% were male. The most common underlying liver diseases were viral hepatitis C (HCV: 49%), and hepatitis B (HBV: 27%). At listing, 84% of patients met the Milan criteria, and 78% received bridging therapy. By the time of transplant, 91% were within the Milan criteria. The median MELD score was 10 (IQR: 8-14), median AFP level was 8 ng/mL (IQR: 4-25), largest tumor size was 2 cm (IQR: 1.1-3.0), and median tumor count was 1 (IQR: 1-2). 24% of patients received living donor grafts, while the remainder received deceased donor grafts. On explant pathology, 71% of patients were within the Milan criteria, 11% had poorly differentiated tumors, and 20% had microvascular invasion.

Compared to the development cohort (n = 739), the validation cohort had a higher prevalence of HBV (27% vs. 21%, *p* < 0.001) and a greater proportion of patients within the Milan criteria at listing (84% vs. 69%, *p* < 0.001), at transplant (91% vs. 79%, *p* < 0.001), and on explant pathology (71% vs. 50%, *p* < 0.001). While the median MELD score at transplant was similar (10 in both cohorts), AFP levels were lower in the validation cohort (8 ng/mL [IQR: 4–25] vs. 9 ng/mL [IQR: 5–34], *p* < 0.001). Tumor size (2 cm [IQR: 1–3] vs. 3 cm [IQR: 2–4], *p* < 0.001) and tumor number (2 [IQR: 1–3] vs. 2 [IQR: 1–4], *p* < 0.001) on explant pathology were also lower in the validation cohort. However, the percentage of poorly differentiated tumors was higher (11% vs. 8%, *p* < 0.001), while the occurrence of microvascular invasion was lower (20% vs. 28%, *p* < 0.001) (Table 2).

**Table 2:** Patient demographics and tumor characteristics of the validation and development cohorts

Variables	Validation (*n* = 2844)	Development (*n* = 739)	*p*-value
Male sex, *n* (%)	2270 (79.8)	612 (82.8)	0.075
Age (years), median (IQR)	59.0 (54.0–64.0)	58.8 (53.0–63.0)	0.021
BMI (kg/m2), median (IQR)	26.1 (23.5–29.4)	26.7 (24.2–30.1)	<0.001
Etiology, n (%)			<0.001
HBV	778 (27.4)	156 (21.1)	
HCV	1389 (48.8)	371 (50.2)	
ALD	412 (14.5)	103 (13.9)	
MAFLD	149 (5.2)	46 (6.2)	
Other	116 (4.1)	63 (8.5)	
AFP level at listing (ng/mL), median (IQR)	10 (4–33)	10 (5–39)	0.003
Tumor size at listing (cm), median (IQR)	2.5 (2.0–3.4)	2.7 (1.8–3.8)	0.034
Tumor number at listing, median (IQR); mean (SD)	1 (1–2);1.6 (1.0)	1 (1–2);2.0 (1.7)	<0.001
Within Milan criteria at listing (%)	2297 (83.6)	509 (68.9)	<0.001
Number of bridging therapies, n (%)			<0.001
0	619 (21.8)	256 (34.6)	
1	939 (33.0)	307 (41.5)	
2	732 (25.7)	119 (16.1)	
3	302 (10.6)	42 (5.7)	
4	118 (4.1)	13 (1.8)	
≥5	134 (4.7)	2 (0.3)	
MELD score before LT, median (IQR)	10 (8–14)	10 (8–14)	0.927
AFP level before LT (ng/mL), median (IQR)	8 (4–25)	9 (5–34)	<0.001
Tumor size before LT (cm), median (IQR)	2.0 (1.1–3.0)	1.6 (0.0–3.0)	<0.001
Tumor number before LT, median (IQR); mean (SD)	1 (1–2);1.5 (1.2)	1 (0–2);1.4 (1.6)	<0.001
Within Milan criteria before LT (%)	1935 (91.4)	580 (78.5)	<0.001
Living donor liver graft, n (%)	683 (24.0)	142 (19.2)	0.007
Tumor size on pathology (cm), median (IQR)	2.0 (1.0-3.0)	3.0 (2.0–4.0)	<0.001
Tumor number on pathology, median (IQR); Mean (SD)	2 (1–3);2.3 (2.9)	2 (1–4);4.0 (8.9)	<0.001
Within Milan criteria on pathology, n (%)	1468 (70.6)	368 (49.9)	<0.001
Tumor differentiation, n (%)			<0.001
Poor	301 (10.6)	57 (7.7)	
Moderate	1431 (50.3)	457 (61.8)	
Well	434 (15.3)	89 (12.0)	
Necrosis	337 (11.8)	45 (6.1)	
Not assessed	341 (12.0)	91 (12.3)	
Microvascular invasion, n (%)	526 (19.6)	207 (28.0)	<0.001

*AFP* Alpha-fetoprotein, *ALD* Alcohol-associated Liver Disease, *BMI* Body Mass Index, *HBV* Hepatitis B Virus, *HCV* Hepatitis C Virus, *IQR* Interquartile Range, *LT* Liver Transplantation, *MAFLD* Metabolic Dysfunction-Associated Fatty Liver Disease, *MELD* Model for End-Stage Liver Disease, *SD* Standard Deviation.

### Tumor recurrence as the event of interest

**Fig. 1:**
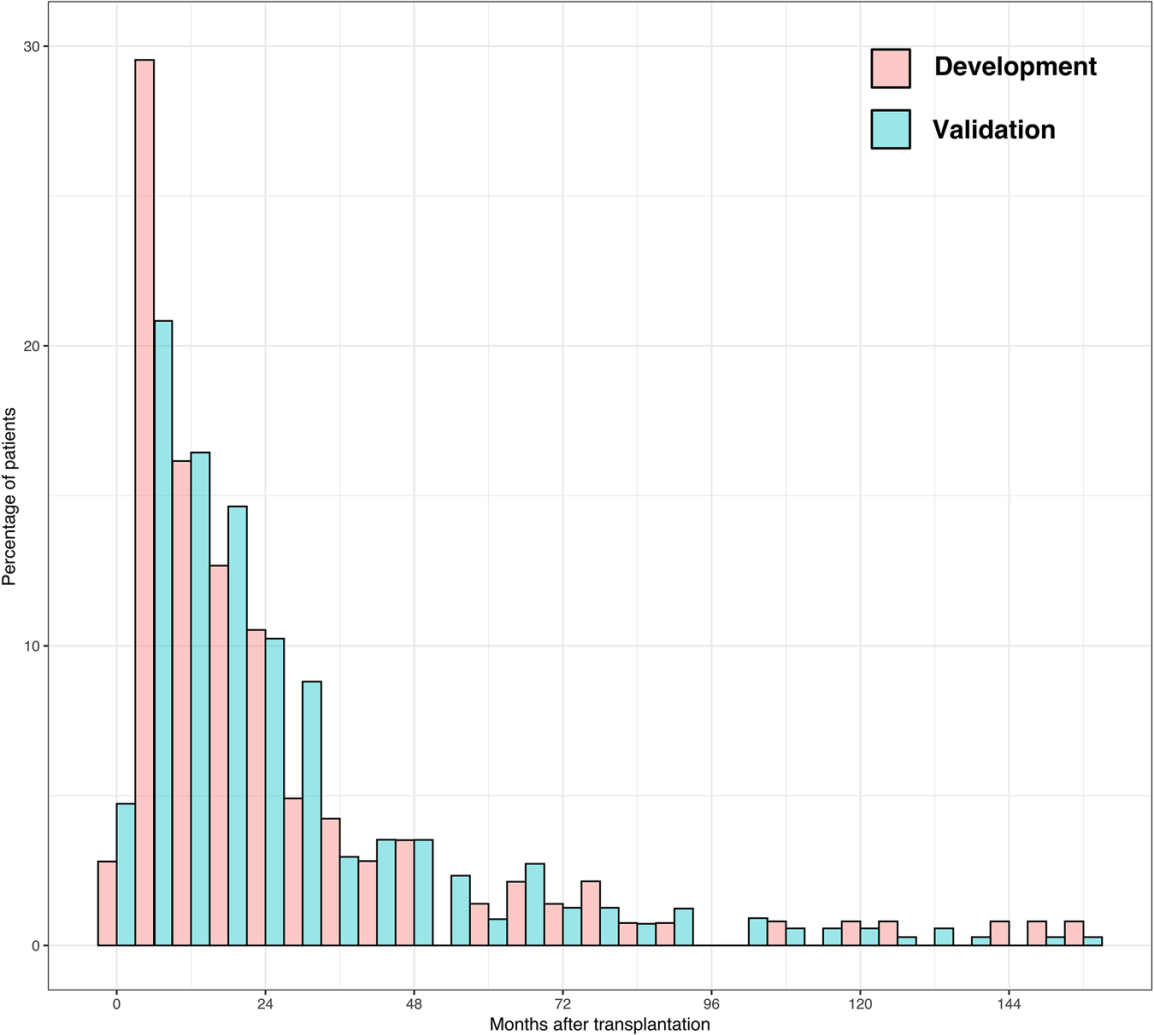
Distribution of recurrences over time (months). Histogram showing the percentage of patients who developed HCC recurrence over time (in months) after liver transplantation. Pink bars represent the development cohort, and turquoise bars represent the validation cohort.

**Fig. 2:**
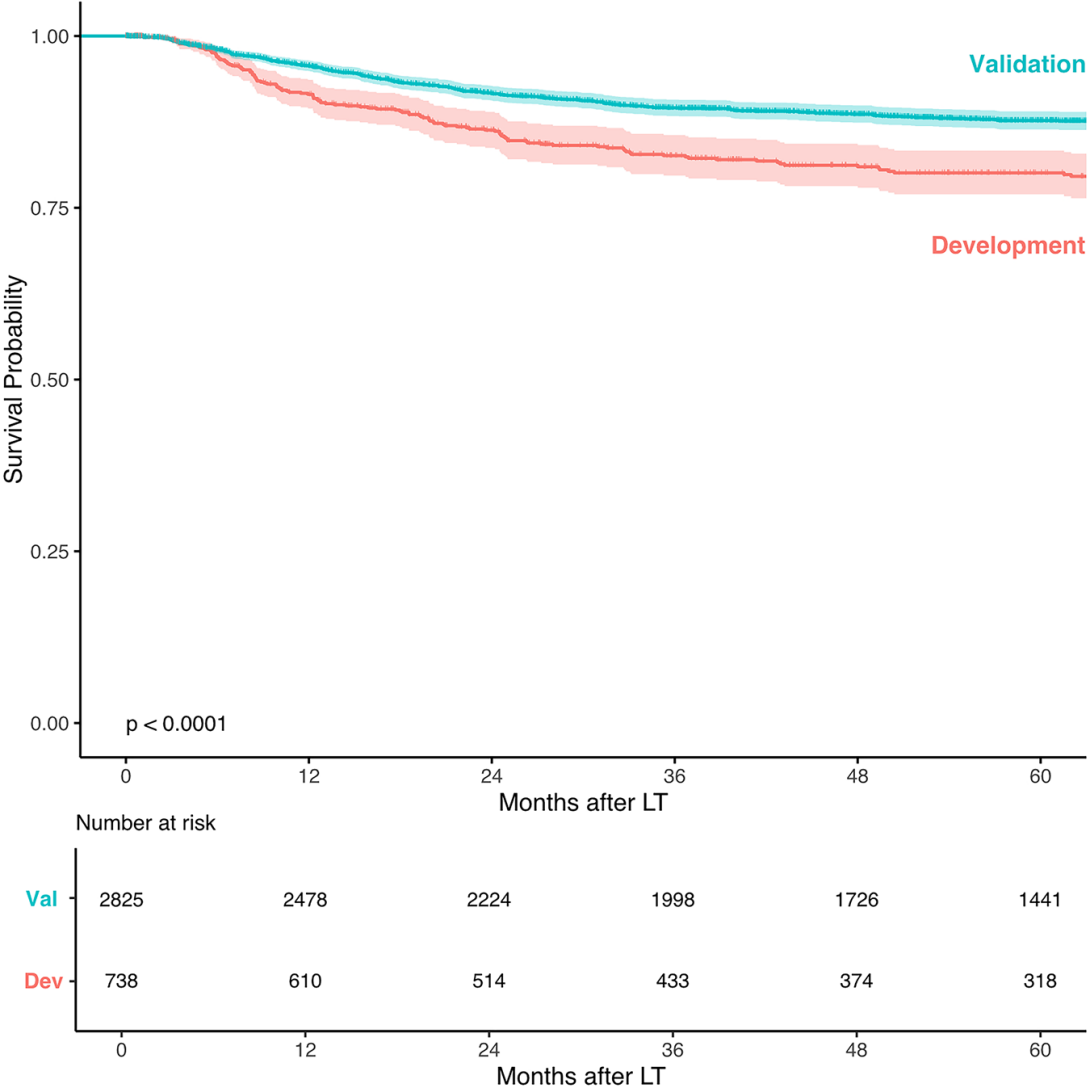
Recurrence-free survival in the validation and development cohorts. Kaplan–Meier curves depicting recurrence-free survival following liver transplantation in the development (pink) and validation (turquoise) cohorts. Comparison was performed using the log-rank test. Dev Development cohort, Val Validation cohort, LT Liver transplantation.

The median follow-up was 5.7 years (IQR: 3.3–9.5) in the validation cohort and 5.5 years (IQR: 2.4–10.2) in the development cohort. In the validation cohort, the overall recurrence rate was 9.1%, lower than the 19.4% observed in the development cohort. The median time from LT to tumor recurrence was 17.5 months (IQR: 8.9–32.3) in the validation cohort, compared to 16.2 months (IQR: 8.2–32.4) in the development cohort. Figure 1 shows the distribution of recurrence over time for both cohorts. Recurrence-free survival rates in the validation cohort were 95.7%, 89.5%, and 87.7% at 1, 3, and 5 years, respectively, compared to 91.5%, 82.6%, and 80.1% in the development cohort (log-rank test, *p* < 0.001) (Fig. 2).

### Comparison of Model Performance

**Fig. 3:**
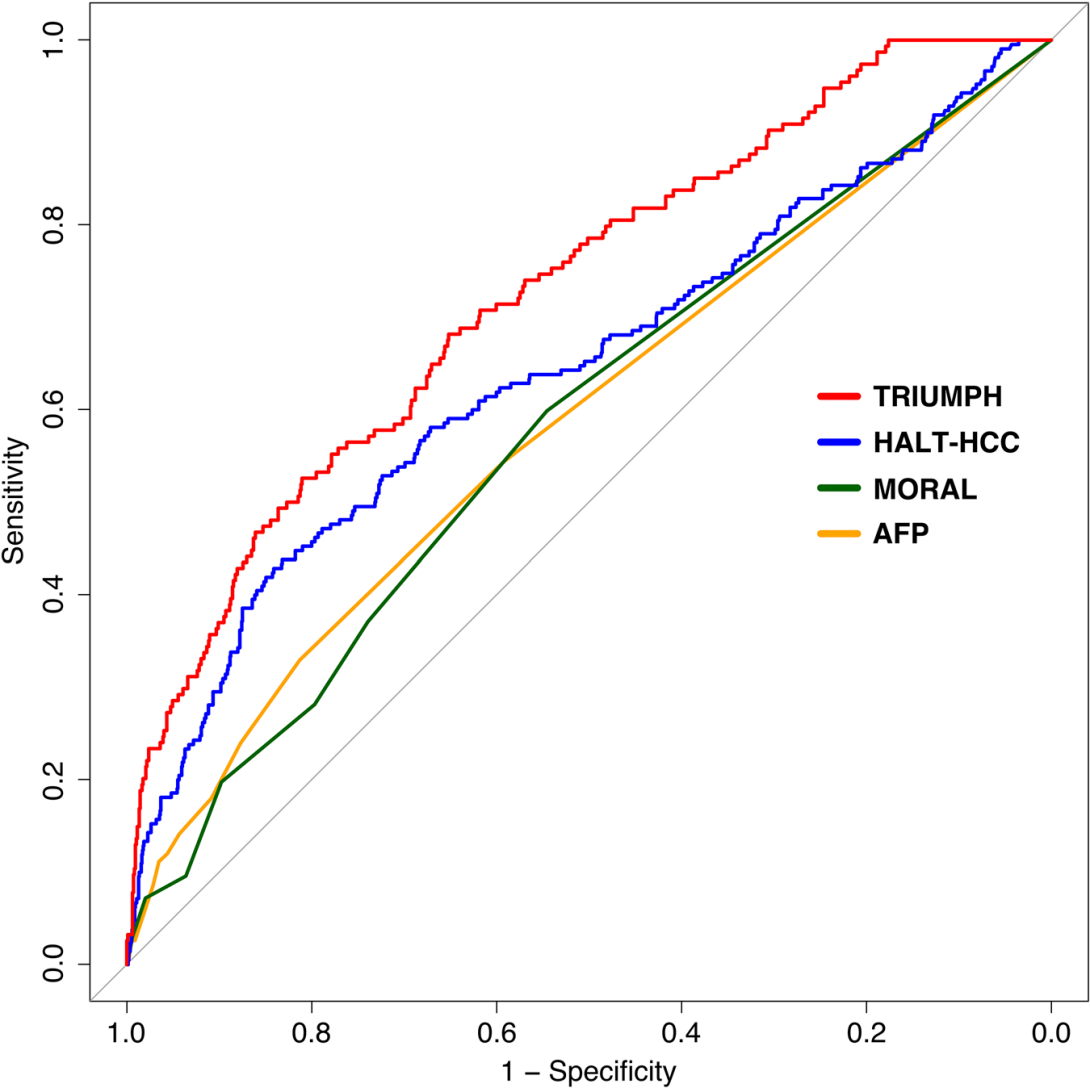
Receiver operating characteristic comparison across the four models. Receiver operating characteristic (ROC) curves comparing the predictive performance of four models: TRIUMPH (red), HALT-HCC (blue), MORAL (green), and AFP (yellow).

The TRIUMPH model achieved the highest c-index at 0.71 (95% CI: 0.62–0.79), though this was not statistically superior to the HALT-HCC model (c-index: 0.67, 95% CI: 0.59–0.75, *p* = 0.28). However, TRIUMPH outperformed both the MORAL (c-index: 0.61, 95% CI: 0.52–0.69, *p* = 0.049 and AFP (c-index: 0.61, 95% CI: 0.53–0.68, *p* = 0.04) models (Table 3). Figure 3 presents the ROC curves for all four models. Supplementary Fig. 1 shows the calibration curve for the TRIUMPH model.

**Table 3:** Comparison of the model’s discriminatory performance

	c-index (95%CI)	z-value	p-value
Overall: TRIUMPH	0.71 (0.62–0.79)		
Overall: HALT-HCC	0.67 (0.59–0.75)	0.59	0.28
Overall: MORAL	0.61 (0.52–0.69)	1.67	0.05
Overall: AFP	0.61 (0.53–0.68)	1.79	0.04
DDLT: TRIUMPH	0.73 (0.63–0.82)		
DDLT: HALT-HCC	0.70 (0.61–0.79)	0.36	0.36
DDLT: MORAL	0.64 (0.55–0.74)	0.2	0.12
DDLT: AFP	0.62 (0.54–0.70)	1.61	0.05
LDLT: TRIUMP	0.66 (0.49–0.83)		
LDLT: HALT-HCC	0.58 (0.41–0.76)	0.58	0.28
LDLT: MORAL	0.52 (0.37–0.66)	1.25	0.11
LDLT: AFP	0.56 (0.42–0.70)	0.85	0.20
Kaohsiung: TRIUMPH	0.63 (0.44–0.82)		
Kaohsiung: HALT-HCC	0.58 (0.39–0.77)	0.37	0.36
Kaohsiung: MORAL	0.52 (0.36–0.67)	0.92	0.18
Kaohsiung: AFP	0.54 (0.40–0.69)	0.69	0.25
Niguarda & Sapienza & Brussels: TRIUMPH	0.77 (0.67–0.87)		
Niguarda & Sapienza & Brussels: HALT-HCC	0.70 (0.60–0.79)	1.03	0.15
Niguarda & Sapienza & Brussels: MORAL	0.64 (0.52–0.75)	1.68	0.05
Niguarda & Sapienza & Brussels: AFP	0.61 (0.51–0.71)	2.24	0.01
UCSF & UCLA: TRIUMPH	0.72 (0.57–0.87)		
UCSF & UCLA: HALT-HCC	0.74 (0.58–0.91)	−0.24	0.59
UCSF & UCLA: MORAL	0.65 (0.49–0.80)	0.65	0.26
UCSF & UCLA: AFP	0.66 (0.52–0.79)	0.61	0.27

*CI* Confidence Interval, *DDLT* Deceased Donor Liver Transplant, *LDLT* Living Donor Liver Transplant, *UCLA* University of California, Los Angeles, *UCSF* University of California, San Francisco.

In subgroup analyses, TRIUMPH consistently showed higher c-index values in patients who received deceased donor LT (c-index: 0.73, 95% CI: 0.63–0.82) and living donor LT (LDLT) (c-index: 0.66, 95% CI: 0.49–0.83) than the three comparator models. Performance varied by region. In the Asian center (Kaohsiung Chang Gung Memorial Hospital), where most transplants were LDLTs, TRIUMPH numerically had the highest c-index of 0.63 (95% CI: 0.44–0.82), compared other models. In European centers (Niguarda Ca’ Granda Hospital, Sapienza University of Rome, Université catholique de Louvain), TRIUMPH achieved a high c-index of 0.77 (95% CI: 0.67–0.87), statistically superior to the MORAL and AFP models. In North American centers (UCSF, UCLA), the c-index of TRIUMPH was 0.72 (95% CI: 0.57–0.87), slightly lower than HALT-HCC (c-index: 0.74, 95% CI: 0.58–0.91) but still higher than the MORAL and AFP models. Patient and tumor characteristics across the three regions (Asia, Europe, North America) are summarized in Supplementary Data 1.

### Net Benefit Analysis

**Fig. 4:**
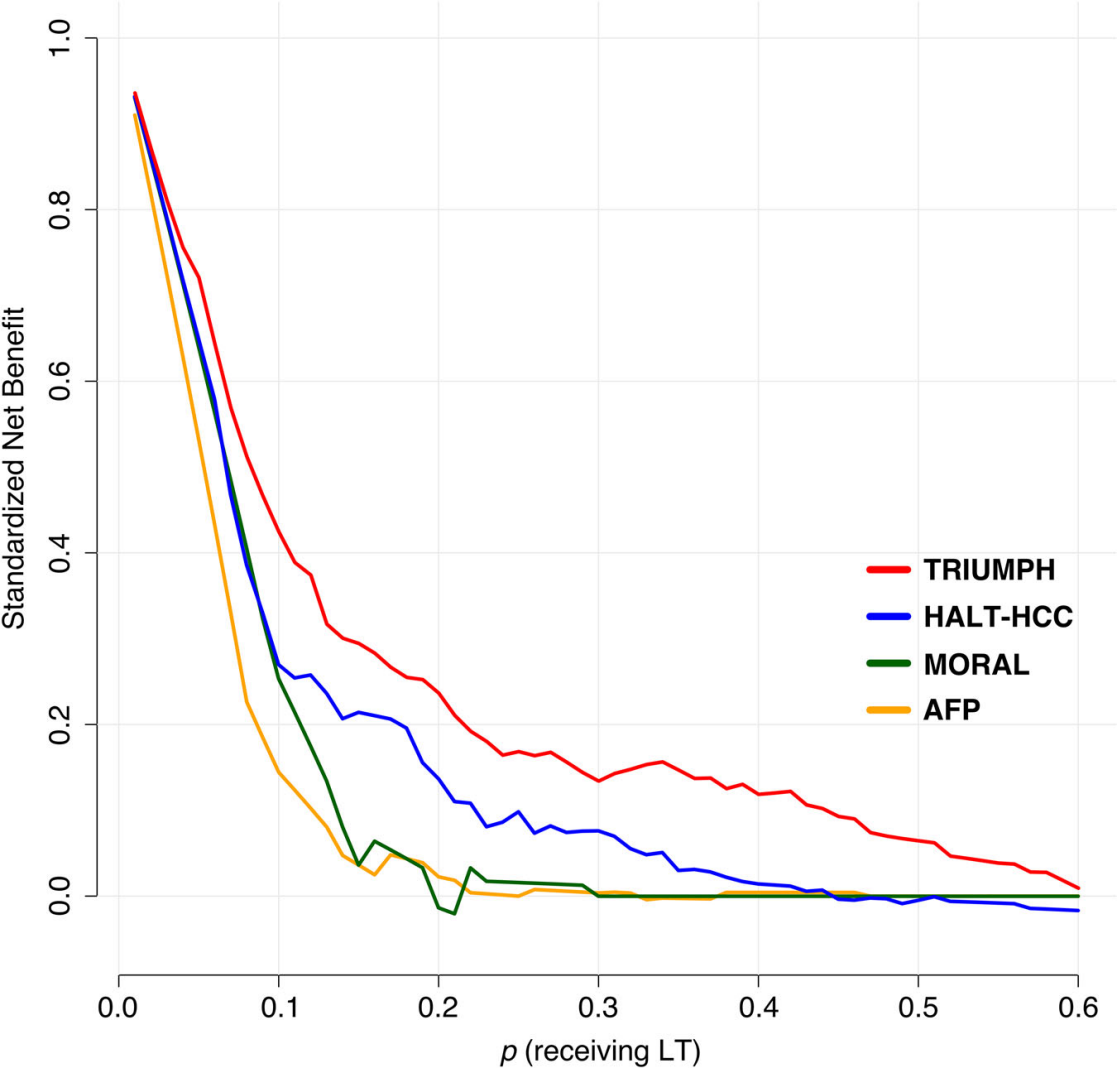
Net benefit decision curve analysis of the four models. Decision curve analysis demonstrating the net benefit of four predictive models—TRIUMPH (red), HALT-HCC (blue), MORAL (green), and AFP (yellow)—across a range of threshold probabilities for selecting patients for liver transplantation (LT).

Net benefit decision curve analysis assesses a model’s clinical value by weighing the benefits of true positives against the harms of false positives, considering the threshold probability at which clinical decisions (e.g., treatment) are made. In this case, the analysis evaluated the usefulness of recurrence risk prediction models for determining liver allograft allocation. The threshold probability reflected organ availability: when no organs were available (probability = 0) or when supply met demand (probability = 1), the models became irrelevant. Figure 4 shows the net benefit decision curve for four models and highlights that the TRIUMPH model consistently provided the highest net benefit compared to HALT-HCC, MORAL, and AFP across a wide range of threshold probabilities. While all models performed similarly at lower thresholds (below 0.1), TRIUMPH maintained a clear advantage up to a threshold of 0.6, where its clinical utility remained superior. HALT-HCC showed intermediate performance, while MORAL and AFP provided the lowest net benefit, particularly as the threshold increases. Overall, TRIUMPH was the most beneficial model for guiding liver transplant allocation decisions, especially in scenarios with moderate organ availability.

## Discussion

We validated our TRIUMPH model, developed using a machine learning approach, in an international multicenter external cohort. The model showed numerically superior discrimination compared to the HALT-HCC, MORAL, and AFP models and had greater clinical utility in the net benefit analysis. These findings support the use of machine learning to create more precise risk prediction models.

The TRIUMPH model’s strength comes from two key factors. First, it was developed using a large LT cohort from the University Health Network, which included nearly 20% LDLT^15^. In contrast, the HALT-HCC, MORAL, and AFP models were based solely on deceased donor LT cohorts. Incorporating a large LDLT population in model development supports a more balanced evaluation of LT recipients and enhances generalizability. This is reflected in TRIUMPH’s numerically higher performance in both the LDLT subgroup and the Kaohsiung Chang Gung Memorial Hospital subgroup. Second, the machine learning approach used in the TRIUMPH model identified more associations and incorporated a broader range of risk factors. It included not only morphological factors like the size and number of lesions but also biomarkers such as AFP levels and neutrophil counts. Additionally, it accounted for dynamic changes occurring during bridging therapies on the waitlist. Incorporating multiple aspects of HCC is crucial for establishing effective selection criteria^16^. Compared to the TRIUMPH model, only HALT-HCC^7^ achieved statistically noninferior performance, consistent with the original development cohort findings. Although this difference wasn’t statistically significant, subgroup analysis showed a numerical advantage for TRIUMPH, particularly among LDLT recipients and non-US cohorts. HALT-HCC, developed at a US center (Cleveland Clinic), performed best in US validation centers (UCSF, UCLA). This highlights the influence of regional practices and patient characteristics on the model’s performance. Despite both models originating from North American centers, TRIUMPH’s superior performance outside North America may stem from its more diverse development cohort and machine learning approach incorporating more predictors.

The TRIUMPH model also showed better clinical utility in the net benefit decision analysis which balanced the true positive rate (preventing futile liver transplants in patients likely to experience recurrence) against the false positive rate (denying transplants to patients who could potentially be cured). Across various risk thresholds (reflecting the probability of LT based on waitlist status and organ availability), the TRIUMPH model achieved a higher net benefit than other models, particularly from thresholds 0.0 to 0.6. This range is realistic given that the literature reports a 1-year probability of 54% and an overall probability of 60–73% for LT in patients with HCC on the UNOS waitlist^18–20^.

One example of machine learning implemented in organ allocation is the Optimized Prediction of Mortality (OPOM), evaluated by OPTN/UNOS^21^. While OPOM was designed to improve risk stratification for HCC patients with exception points^22,23^, it only predicts waitlist dropout, neglecting the critical prognostic factor of post-transplant survival. The TRIUMPH model, however, shows strong performance and utility in predicting post-transplant recurrence. Therefore, TRIUMPH could serve as a complementary tool, integrating this crucial post-transplant survival aspect into the organ allocation system. Nevertheless, incorporating any new model like TRIUMPH into allocation policy is a major undertaking that remains a future goal, requiring extensive validation, logistical considerations, and consensus within the transplant community.

Apart from the TRIUMPH model, three other machine learning-based models have been developed to predict post-transplant HCC recurrence: MoRAL-AI^24^, RELAPSE^25^, and TRAIN-AI^26^. The MoRAL-AI model, which uses a deep neural network, incorporates variables such as tumor diameter, AFP, and PIVKA-II. It demonstrated improved discrimination compared to the conventional MoRAL model, however, its generalizability is limited due to its focus on LDLT recipients in South Korea and the requirement for PIVKA-II, which is not routinely analyzed. The RELAPSE model, which employs random survival forests and classification techniques, achieved a higher c-index compared to the TRIUMPH model, although this advantage likely stems from its inclusion of post-transplant variables. However, pre-transplant variables are crucial for decision-making regarding organ allocation, as they are the only factors available before surgery. TRAIN-AI, developed on a large international cohort and validated on a smaller North American cohort, followed the opposite approach of the TRIUMPH model. While TRIUMPH captured dynamic changes in tumor lesions during bridging therapy using objective data, TRAIN-AI relied on the modified Response Evaluation Criteria in Solid Tumors (mRECIST). This criterion, which can vary between institutions and radiologists^27,28^, may introduce bias. Nevertheless, TRAIN-AI achieved a high c-index of 0.77 in both internal and external validations, outperforming other pre-existing models. The DeepSurv methodology, used by TRAIN-AI, was also considered for our development cohort. However, the TRIUMPH approach—enhancing the traditional Cox model with elastic net regularization—was preferred due to its superior performance. While DeepSurv is well-suited for large development cohorts like that used in TRAIN-AI, its complexity poses a risk of overfitting in smaller datasets. Given our relatively small sample size, we selected the TRIUMPH model as a more appropriate approach to mitigate this risk.

This study faces several limitations that impact its findings. Firstly, its design as a retrospective and multicentric study introduces the possibility of selection biases which may arise due to the varied approaches to management across different centers. Additionally, the development cohort from Toronto differed significantly from the validation cohort, which had a higher HBV prevalence and lower proportion of locally advanced HCC. This discrepancy may have negatively impacted the model’s validation performance and highlighted potential regional biases that affected the model’s performance. Future work incorporating data from diverse international centers during model development would help refine the model and improve its generalizability. While the inclusion of both living and deceased donor grafts in the TRIUMPH model could be viewed as a commingling of data, this approach was intentional. Donor-specific models may better capture differences in transplant settings or graft characteristics, but the TRIUMPH model was designed to reflect the reality of centers offering both types of transplantation. It provides a unified tool to guide decision-making for patients eligible for either pathway. Notably, there is no evidence to date that graft quality directly influences oncologic outcomes in an as-treated analysis. Given that models specific to LDLT or DDLT already exist, the strength of TRIUMPH lies in its validated performance across a mixed-donor cohort—reflecting real-world practice and enhancing generalizability across diverse transplant programs. Finally, while other frequently used models, such as Metroticket 2.0^29^ (based on competing risk analysis) and RETREAT^14^ (which incorporates pathological predictors), could have been included as comparators, their inclusion was not feasible due to unmet model requirements.

In conclusion, the TRIUMPH model outperforms other commonly used scores in predicting post-transplant HCC recurrence, offering both higher accuracy and greater clinical utility. This suggests that integrating the TRIUMPH model into future organ allocation strategies for HCC patients could enhance the overall benefit of liver transplantation. Our study highlights the potential of machine learning approaches to advance organ allocation in transplant medicine. However, despite technological advancements, it remains essential to develop robust machine learning models with large, diverse cohorts to ensure generalizability and avoid overfitting. This will require ongoing collaboration within the international transplant community and a commitment to incorporating machine learning innovations into transplant practices.

## Supplementary information

Supplemental Information

Description of Additional Supplementary Files

Supplemental Data 1

Reporting summary

## Data Availability

The clinical datasets generated and analyzed during the current study are not publicly available due to their containing sensitive, protected health information (PHI). As stipulated by the University Health Network’s Institutional Review board (IRB) and in accordance with the patient consent forms, which do not permit public data deposition, the raw data cannot be shared in a public repository. However, a de-identified dataset may be made available to qualified researchers for verification or secondary analysis upon reasonable request. Access is subject to a formal data use agreement (DUA) and approval from the original IRB. Interested parties may contact the corresponding author to initiate a request.

## Code availability

All code used for data analysis in this study is available in the supplementary materials.
