# Supplementary material for: Validation of the Toronto recurrence inference using machine-learning for post-transplant hepatocellular carcinoma model
Source: Commun Med (Lond). 2025 Jul 9;5:284. doi: 10.1038/s43856-025-00994-5 (PMC12238485; doi:10.1038/s43856-025-00994-5)
Supplement: Supplementary file 1 — Supplemental Information [file 43856_2025_994_MOESM1_ESM.pdf]

Supplementary Figure 1: Calibration curve for the TRIUMPH model. Calibration curve comparing predicted versus observed survival probabilities. The black line represents the model's predicted calibration, while the red diagonal line indicates perfect agreement (ideal reference line).

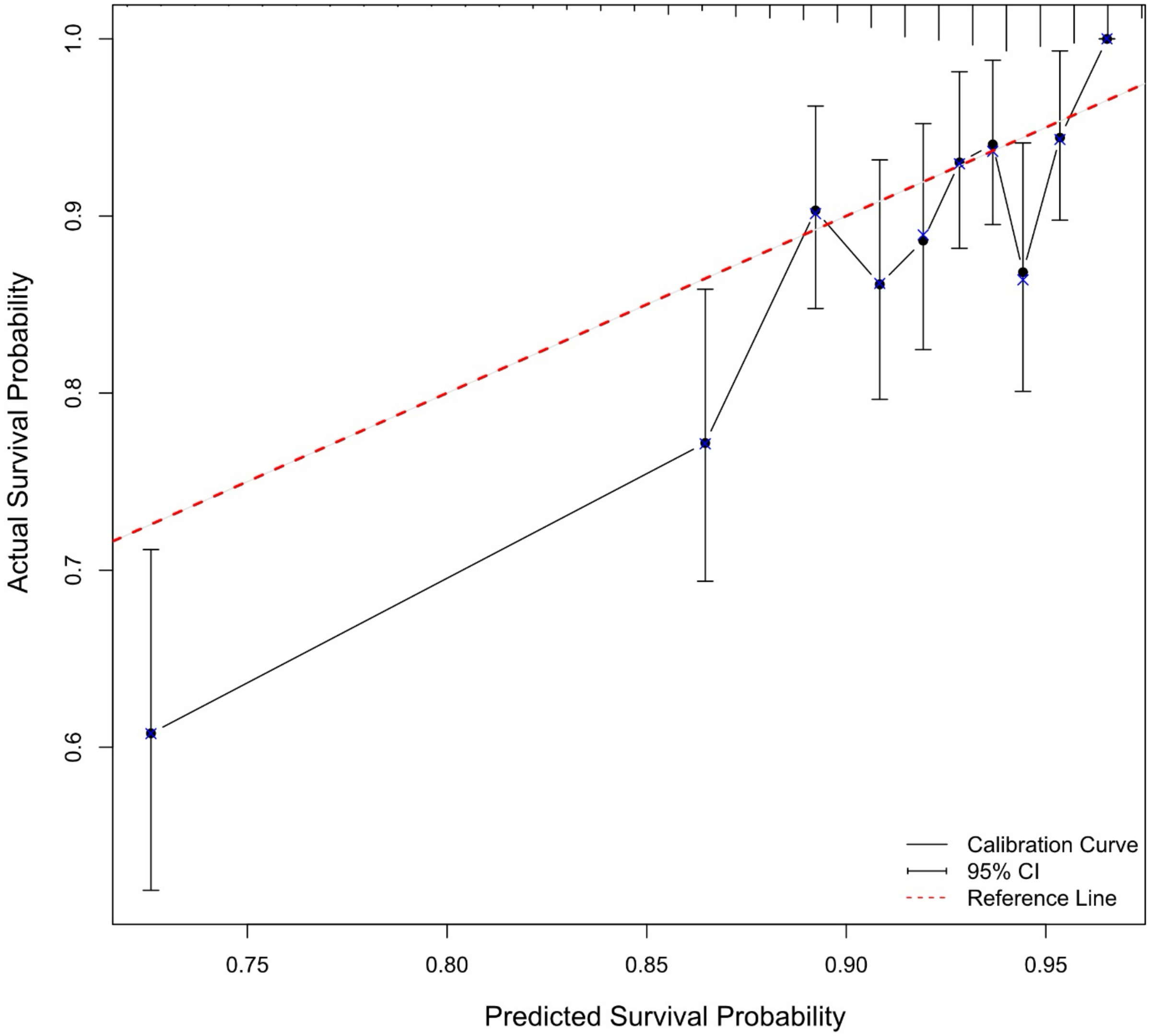

R Scripts Used in Data Analysis

```
# R Script for Survival Analysis and Model Validation
# Author: Zhihao Li
# Date: 2025-06-07
#
# Description:
# This script performs survival analysis and validates several predictive models
# for hepatocellular carcinoma recurrence after liver transplantation. It includes:
# 1. Data loading and preparation.
# 2. Generation of descriptive statistics (Table 1).
# 3. Kaplan-Meier survival analysis.
# 4. Evaluation of predictive models based on:
#    - Discrimination (Concordance Index)
#    - Calibration (Calibration Plots)
#    - Clinical Utility (Decision Curve Analysis)

# ---
# Section 1: Setup - Load Libraries and Data
# ---

# Load required packages
library(readxl)     # For reading Excel files
library(dplyr)      # For data manipulation
library(tableone)   # For creating patient characteristics tables
library(survival)   # For survival analysis core functions
library(survminer)  # For plotting Kaplan-Meier curves
library(rms)        # For calibration plots (requires Hmisc)
library(rmda)       # For decision curve analysis
library(ggplot2)    # For advanced plotting

# --- Set Working Directory ---
# It's recommended to use RStudio Projects to manage working directories.
# Otherwise, uncomment and set your directory path below.
# setwd("path/to/your/project/folder")

# --- Load Datasets ---
# Load data for Table 1 and survival analysis
df_tableone <- read_xlsx("validation_data.xlsx") %>% as.data.frame()
df_surv <- read_xlsx("dfsurv.xlsx") %>% as.data.frame()

# Load data for model validation
df_validation <- read_xlsx("validation_data2.xlsx") %>% as.data.frame()
```

```
# ---
# Section 2: Descriptive Statistics (Table 1)
# ---

# Define variable lists for the table
cat_vars <- c("male", "etiologysorted", "listing_milan", "bridging_y",
           "bridging_number", "pretx_milan", "transplant_procedure",
           "transplant_path_milan", "transplant_path_differentiation",
           "transplant_microvascular_invasion")

all_vars <- c("male", "age", "bmi", "listing_meld", "listing_afp",
           "etiologysorted", "listing_lesion_size", "listing_lesion_number",
           "listing_milan", "bridging_y", "bridging_number", "pretx_lesion_size",
           "pretx_lesion_number", "pretx_milan", "transplant_procedure",
           "transplant_path_milan", "transplant_path_tumor_size",
           "transplant_path_tumor_number", "transplant_path_differentiation",
           "transplant_microvascular_invasion", "pretx_meld", "pretx_afp")

# Create the summary table, stratified by medical center
table1 <- CreateTableOne(vars = all_vars,
                         strata = "center",
                         data = df_tableone,
                         factorVars = cat_vars)

# Print and export the table to a CSV file
table1_matrix <- print(table1,
                       quote = FALSE,
                       noSpaces = TRUE,
                       printToggle = FALSE,
                       showAllLevels = TRUE)

write.csv(table1_matrix, file = "Table1_Descriptive_Statistics.csv")


# ---
# Section 3: Recurrence-Free Survival Analysis
# ---

# Fit Kaplan-Meier survival model
# 'recurfreesurv' is in days, converted to months for the plot
km_fit <- survfit(Surv(recurfreesurv / 30, recur) ~ c, data = df_surv)

# Plot Kaplan-Meier curve with risk table
ggsurvplot(
```

```r
  fit = km_fit,
  legend.title = element_blank(),
  conf.int = TRUE,
  pval = TRUE,
  pval.coord = c(0, 0),
  risk.table = TRUE,
  xlim = c(0, 60),
  break.time.by = 12,
  surv.median.line = "hv",
  xlab = "Months after Liver Transplantation",
  ylab = "Recurrence-Free Survival Probability",
  ggtheme = theme_classic(base_size = 16),
  conf.int.style = "ribbon"
)

# Save the plot
ggsave("Kaplan_Meier_Plot.png", width = 10, height = 8)

# ---
# Section 4: Predictive Model Evaluation
# ---

# --- 4.1 Data Preparation for Validation ---

# Convert follow-up dates and calculate survival time in days
df_validation$fu_date <- as.Date(df_validation$fu2, format = "%d.%m.%Y")
df_validation$txdate <- as.Date(df_validation$txdate, format = "%d.%m.%Y")
df_validation$recurfreesurv <- as.numeric(difftime(df_validation$fu_date, df_validation$txdate,
units = "days"))

# Feature Engineering: Create predictors required by the models
df_validation <- df_validation %>%
  mutate(
    # General predictors
    tbs = sqrt(pretx_lesion_size^2 + pretx_lesion_number^2),
    etiology_other = ifelse(etiology == "Other", 1, 0),

    # Predictors for MORAL score
    afpmax200 = ifelse(afp_max >= 200, 1, 0),
    nlr5 = ifelse(pretx_nlr >= 5, 1, 0),
    size3 = ifelse(pretx_lesion_size >= 3, 1, 0),

    # Predictors for AFP score
    sizelist36 = ifelse(listing_lesion_size > 3 & listing_lesion_size <= 6, 1, 0),
```

```
    sizelist6 = ifelse(listing_lesion_size > 6, 1, 0),
    countlist4 = ifelse(listing_lesion_number >= 4, 1, 0),
    afplist1000low = ifelse(listing_afp > 100 & listing_afp <= 1000, 1, 0),
    afplist1000high = ifelse(listing_afp > 1000, 1, 0)
  )

# --- 4.2 Calculate Prognostic Index (PI) for Each Model ---
df_validation <- df_validation %>%
  mutate(
    # Note: Coefficients are taken from the original publications.
    # Ensure these are the correct, final coefficients for your analysis.
    pi_coxnet = (age * -0.004) + (nrBT * 0.228) + (etiology_other * -0.153) +
                (listing_diameter * 0.041) + (pretx_lesion_size * 0.02) +
                (listing_lesion_size * 0.02) + (log(pretx_afp) * 0.191) +
                (pretx_milan * -0.06) + (pretx_neutro * 0.025) +
                (pretx_sodium * -0.01) + (tbs * 0.038),

    pi_halthcc = (log(pretx_afp) * 0.547) + (tbs * 0.376) + (pretx_meld * 0.077),

    pi_moral = (afpmax200 * 0.318) + (nlr5 * 0.417) + (size3 * 0.265),

    pi_afp = (sizelist36 * 0.069) + (sizelist6 * 0.343) + (countlist4 * 0.177) +
             (afplist1000low * 0.170) + (afplist1000high * 0.241)
  )

# --- 4.3 Discrimination: Concordance Index (C-Index) ---

# Calculate C-Index for all models
# The `rcorr.cens` function from the Hmisc package is used.
# Note: A higher PI score should correspond to a worse outcome, so we use -PI.
c_index_results <- list(
  "CoxNet" = Hmisc::rcorr.cens(-pi_coxnet, Surv(recurfreesurv, recur)),
  "HaltHCC" = Hmisc::rcorr.cens(-pi_halthcc, Surv(recurfreesurv, recur)),
  "MORAL" = Hmisc::rcorr.cens(-pi_moral, Surv(recurfreesurv, recur)),
  "AFP Score" = Hmisc::rcorr.cens(-pi_afp, Surv(recurfreesurv, recur))
)

# Extract and print C-indices
c_indices <- sapply(c_index_results, function(x) x["C Index"])
print(c_indices)

# Example: Compare C-index of two models (e.g., CoxNet vs. MORAL)
c_index_model1 <- c_index_results$CoxNet["C Index"]
se_model1 <- c_index_results$CoxNet["S.D."] / 2 # rcorr.cens gives S.D. for 2*C
```

```
c_index_model2 <- c_index_results$MORAL["C Index"]
se_model2 <- c_index_results$MORAL["S.D."] / 2

# Two-sided z-test for comparing C-indices
z_stat <- (c_index_model1 - c_index_model2) / sqrt(se_model1^2 + se_model2^2)
p_value <- 2 * pnorm(-abs(z_stat))
cat(sprintf("Comparison of C-indices: Z-statistic = %.3f, P-value = %.4f\n", z_stat, p_value))


# --- 4.4 Calibration Analysis ---

# Set up datadist for rms functions
dd <- datadist(df_validation)
options(datadist = 'dd')

# Fit a Cox model using the AFP score's prognostic index
# x=TRUE and y=TRUE are required for calibration
cox_model_rms <- cph(Surv(recurfreesurv, recur) ~ pi_afp,
                data = df_validation,
                x = TRUE, y = TRUE, surv = TRUE)

# Generate calibration data at a specific time point (e.g., 5 years = 1825 days)
# Use bootstrapping (B > 0) for confidence intervals
cal_data <- calibrate(cox_model_rms,
                 u = 1825, # 5 years
                 method = "boot",
                 B = 500,
                 m = floor(nrow(df_validation) / 3)) # Adjust 'm' based on sample size

# Plot the calibration curve
plot(cal_data,
    xlab = "Predicted 5-Year Survival Probability",
    ylab = "Actual 5-Year Survival Probability (Kaplan-Meier)",
    main = "Calibration Plot for the AFP Score (5-Year)")
abline(0, 1, col = "red", lty = 2) # Add ideal reference line

options(datadist = NULL) # Clean up global option


# --- 4.5 Clinical Utility: Decision Curve Analysis (DCA) ---

# Fit Cox models for each PI to get predicted probabilities for DCA
# The rmda package requires fitting a model first.
fit_coxnet <- coxph(Surv(recurfreesurv, recur) ~ pi_coxnet, data = df_validation)
```

```
fit_halthcc <- coxph(Surv(recurfreesurv, recur) ~ pi_halthcc, data = df_validation)
fit_moral <- coxph(Surv(recurfreesurv, recur) ~ pi_moral, data = df_validation)
fit_afp <- coxph(Surv(recurfreesurv, recur) ~ pi_afp, data = df_validation)

# Create a list of models for plotting
dca_models <- list(
  "CoxNet" = fit_coxnet,
  "HaltHCC" = fit_halthcc,
  "MORAL" = fit_moral,
  "AFP Score" = fit_afp
)

# Generate and plot the decision curves for a specific time point (e.g., 5 years)
plot_decision_curve(dca_models,
                    curve.names = names(dca_models),
                    cost.benefit.axis = FALSE,
                    col = c("blue", "green", "purple", "orange"),
                    confidence.intervals = FALSE, # Set to TRUE for CIs (slower)
                    standardize = FALSE,
                    time = 1825, # 5-year prediction
                    legend.position = "topright") +
  labs(title = "Decision Curve Analysis for 5-Year Recurrence")

ggsave("Decision_Curve_Analysis.png", width = 10, height = 8)
```
