# Supplementary material for: Validation of the Toronto recurrence inference using machine-learning for post-transplant hepatocellular carcinoma model
Source: Commun Med (Lond). 2025 Jul 9;5:284. doi: 10.1038/s43856-025-00994-5 (PMC12238485; doi:10.1038/s43856-025-00994-5)
Supplement: Supplementary file 2 — Description of Additional Supplementary Files [file 43856_2025_994_MOESM2_ESM.pdf]

Description of Additional Supplementary Files

File name: Supplementary Data 1

Description: Patient and tumor characteristics across the three regions (Asia, Europe, North America)
